# Intranasal administration of stem cells and their derivatives for neurological and respiratory disorders: a systematic review of human clinical trials

**DOI:** 10.3389/fnagi.2026.1834543

**Published:** 2026-05-07

**Authors:** Umm E. Habiba, Roshini Sathyanarayanan, Sabiha Shamim, Aishwarya Manian, Ammar Haider, Iqra Sarwar, David Lawrence Greene

**Affiliations:** 1 Research and Development (R&D) Department, R3 Stem Cell LLC, Scottsdale, AZ, United States; 2 Research and Development (R&D) Department, R3 Medical Research LLC, Islamabad, Pakistan

**Keywords:** blood brain barrier, clinical trials, intranasal, mesenchymal stem cells, neural stem cells, neurological disorders

## Abstract

**Objective:**

This systematic review evaluates the safety, feasibility, tolerability, and efficacy outcomes of intranasally administered stem cells and their derivatives (MSCs, NSCs, secretome, and EVs) for the treatment of neurological and respiratory disorders.

**Methods:**

A literature search was conducted across PubMed, Google Scholar, Web of Science, Scopus, and ClinicalTrials.gov for published research from January 2011 to December 2025. A total of 19 studies were included (7 published articles, 12 registry-only or grey literature records). Risk of bias was assessed using two complementary Cochrane tools, the RoB 2 tool for all 19 studies and the ROBINS-I tool for the 6 n-RCTs. Sources of heterogeneity were systematically characterized across 5 clinical dimensions, and structural publication bias was evaluated against a 30% registry-only threshold. A random-effects model was selected for pooled analyses, with a planned subgroup analysis of treatment dosage against adverse events. Effect sizes were extracted using SMD, mean differences, risk ratios, and odds ratios as appropriate per outcome type.

**Results:**

This systematic review was conducted in accordance with PRISMA 2020 guidelines. A total of 104 participants were enrolled in 7 published studies, with one completed registry trial (NCT04602104) confirming actual enrolment of an additional 18 participants. No study achieved an overall low risk of bias under either assessment tool. Under RoB 2, 1 published study was rated as having some concerns, others were rated high risk or some concerns. Under ROBINS-I, 3 n-RCT studies were rated critical overall. The remaining 3 were rated serious. Across the 7 published studies, 98 participants contributed to the analyzed outcomes. Structural publication bias was confirmed. Substantial clinical heterogeneity was identified across 9 conditions, multiple cell product categories, and mixed intranasal and intravenous delivery routes.

**Conclusion:**

Although current evidence suggests that intranasal administration of stem cells, particularly MSCs and NSCs, in humans is a safe and feasible approach, with possible therapeutic improvements in CNS disorders. However, there is a serious risk of bias, critically small sample sizes, and pervasive publication bias in the available literature. Adequately powered, quadruple-blinded, placebo-controlled randomized trials need to be conducted before clinical translation can be considered.

## 1 Introduction

Stem cell-based therapy has emerged as a promising strategy for neurological, neuroinflammatory, and neurodegenerative disorders (Vargas-Rodríguez et al., 2023). These disorders represent a substantial global health burden and are the leading cause of long-term disability and mortality (Owolabi et al., 2023). A major challenge in the management of central nervous system (CNS) disorders is the presence of the blood–brain barrier (BBB), a highly selective, semipermeable membrane that protects the brain from toxins and pathogens by allowing selected, essential substances to pass through (Crowe and Hsu, 2022). The BBB blocks the entry of systemically administered therapeutics, making it a major obstacle to CNS drug delivery.

Traditional routes of stem cell therapy, such as intravenous infusion and intracerebral injection, face significant limitations, including low homing efficiency, invasiveness, and an inability to bypass the BBB (Zhang et al., 2020). Intranasal transport has garnered attention as a non-invasive approach that enables direct delivery of therapeutics, including stem cells, into the CNS (Owolabi et al., 2023; Zhang et al., 2020). The unique anatomical connection between the nasal cavity and the brain (via olfactory and trigeminal nerve branches) provides an excellent opportunity for targeted cell delivery without surgical intervention, thus making it an attractive approach for clinical translation (Wu et al., 2025).

Early human studies have investigated the feasibility of intranasal stem cell transport, including mesenchymal stem cells (MSCs) (Zhang et al., 2020), allogeneic neural stem cells (NSCs) (Lv et al., 2022), and induced pluripotent stem cells (iPSCs) (Li et al., 2024), particularly with MSCs from sources such as bone marrow (Villar-Gómez et al., 2022), umbilical cord (Guo et al., 2021), human breastmilk, and MSC-derived exosomes (Nistor-Cseppentö et al., 2022; Ru et al., 2025; Boika et al., 2020; Wagenaar et al., 2025)More recently, MSC-derived extracellular vesicles (EVs) (Villar-Gómez et al., 2022), including exosomes (Valeri and Mazzon, 2023), have been investigated as a cell-free alternative that retains therapeutic efficacy while reducing potential risks associated with live cell transplantation (Gotoh et al., 2024; Li Y. et al., 2022). In 2024, UCMSC-derived exosomes were administered intranasally to treat motor disorders. No adverse events were observed following treatment, and most patients showed improvement in motor function. The study demonstrated safety and supported the advantage of repeated dosing (Zhao et al., 2023; Prodromos, 2025; Ru et al., 2025; Platt et al., 2020; Vargas-Rodríguez et al., 2023; Qiu et al., 2025; Gotoh et al., 2024; Xie et al., 2023). Recent research discusses intranasal stem cell therapy broadly, but there is a lack of focused systematic synthesis of human clinical studies that critically evaluate safety, feasibility, and efficacy outcomes (Gotoh et al., 2024; Zhang et al., 2021). This systematic review aimed to discuss the existing human evidence, identify knowledge gaps, and outline the types of stem cells used for intranasal (IN) administration, to evaluate whether this approach provides a viable and therapeutic strategy for CNS disorders.

## 2 Materials and methods

### 2.1 Search strategy

A comprehensive and systematic literature search was performed in PubMed, Google Scholar, Web of Science, Scopus, and ClinicalTrials.gov to identify human clinical studies assessing intranasal stem cell administration from January 2011 to December 2025. Search terms consisted of a combination of free-text keywords related to stem cells, intranasal delivery, nose-to-brain route, human subjects, and clinical trials. These keywords were combined using Boolean operators (AND, OR, NOT) to ensure comprehensive retrieval. We used combination of keywords such as (“intranasal” OR “nose-to-brain” OR “nasal administration”) AND (“stem cell” OR “neural stem cell” OR “mesenchymal stem cell” OR “MSC” OR “NSC” OR “umbilical cord stem cell” OR “exosome” OR “cell therapy”) AND (“clinical trial” OR “first-in-human” OR “phase 1” OR “phase 2” OR “Parkinson” OR “cerebral palsy” OR “stroke” OR “perinatal stroke” OR “brain injury” OR “neurological” OR “ENT” OR “HEAD”) NOT (“mouse” OR “mice” OR “rat” OR “rodent” OR “pig” OR “rabbit” OR “primate” OR “animal”). Specific disease and diagnostic keywords (e.g., “Parkinson,” “cerebral palsy,” “stroke,” “perinatal stroke,” and “brain injury”) were deliberately included to ensure that studies with clinically diagnosed patient groups were included.

### 2.2 Eligibility criteria

Criteria for inclusion and exclusion for the articles reviewed in Table 1.

**Table 1:** Criteria for inclusion and exclusion for the articles reviewed.

Item	Inclusion criteria	Exclusion criteria
Population	Participants having neurological or respiratory diseases or healthy volunteers	Participants with diseases other than neurological or respiratory
Intervention	MSCs and/or their derivatives (e.g., MSCs, NSCs and their derived cells, and MSC-derived exosomes, secretomes, and EVs) via intranasal route regardless of their source (autologous/allogeneic/BM/UC/WJ)	Studies using iPSCs and stem cells sourced from breast milk were not included.
Comparator	Studies with or without a comparator were included. Control groups including placebo or sham intranasal sprays; combination with other alternative stem cell delivery routes (e.g., intravenous, intracerebral, intrathecal)	-
Outcome	Reported adverse events linked to treatment OR Clinical improvements in motor, respiratory and non-motor functions. OR Biological findings such as imaging results from MRI, CBC etc.	Studies that did not report adverse events OR clinical improvements
Study design	RCTs, randomized interventional studies, non-randomized controlled trials, pilot studies, prospective and retrospective clinical studies	Case studies, protocols, editorials, conference abstracts, errata, duplicate publications, retracted articles, and articles without full text were excluded. Preclinical (animal and in vivo).

MSC, mesenchymal stem/stromal cells; NSC, neural stem/progenitor cells; EV, extracellular vesicles; RCT, randomized clinical trial; CBC, complete blood count.

### 2.3 Study selection process

Two independent reviewers (DLG and UEH) screened titles and abstracts for relevance. In cases where the two reviewers had differing opinions, a third reviewer (SS) was consulted. Full-text articles were evaluated for eligibility. The process for systematically filtering the research is displayed in a PRISMA flow diagram. Data extraction followed a standardized format, capturing study title, results, design, stem cell type, dose and frequency, follow-up duration, year, population characteristics, and primary and secondary outcomes related to safety and clinical efficacy.

### 2.4 Statistical analysis

Risk of bias was assessed using two complementary Cochrane tools, the Risk of Bias 2 (RoB 2) tool and the Risk of Bias in Non-Randomized Studies of Interventions (ROBINS-I) tool for the non-randomized comparative studies. Sources of heterogeneity were systematically characterized across five clinical dimensions, and structural publication bias was evaluated against a 30% registry-only threshold. A random-effects model was selected for pooled analyses, with a planned subgroup analysis of treatment dosage against adverse events. Effect sizes were extracted using standardized mean differences, mean differences, risk ratios, and odds ratios as appropriate per outcome type.

## 3 Results

### 3.1 Study selection

**Figure 1:**
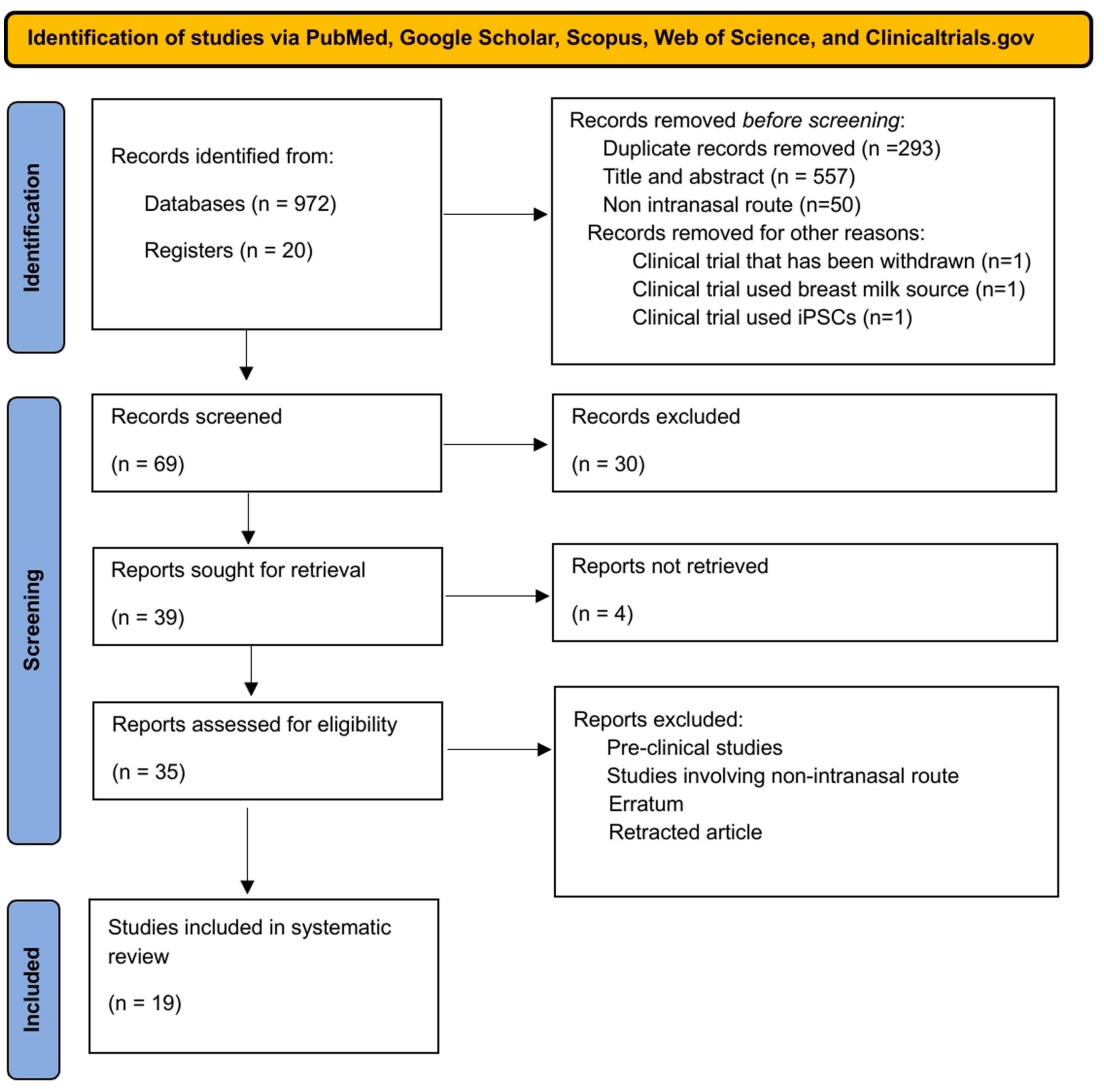
Flow diagram illustrating identification, screening, and selection of the eligible clinical trials/studies for systematic review.

A systematic literature search across databases identified 972 records, of which 679 remained after duplicate removal. Following title and abstract screening, 62 articles underwent full-text assessment and were screened for human trials. After manual removal of animal models, *in vivo* studies, and non-intranasal administration routes, 23 articles were excluded, and the remaining 39 articles were sought for retrieval. Four of these remained inaccessible, leaving 35 studies that met the inclusion criteria for systematic review. From these 35, only 19 studies remained for statistical analysis after in-depth review. The study selection process is illustrated in the PRISMA flow diagram (Figure 1).

### 3.2 PRISMA 2020 adherence

This systematic review was conducted in accordance with PRISMA 2020 guidelines.

### 3.3 Included study attributes

Most of the included trials were early Phase I, Phase I, and Phase I/II clinical trials, with sample sizes varying from 9 to 5,000. Both pediatric and adult populations were included, regardless of gender. Most of the studies focused on neurological conditions, including stroke, cerebral palsy, Parkinson’s disease, dementia, and refractory focal epilepsy, with fewer studies addressing respiratory conditions such as asthma.

Mesenchymal stromal cells (MSCs) occupied a predominant position among cell types utilized; additionally, mesenchymal-derived exosomes and trophic factors were also used. Sources of these cells include bone marrow, umbilical cord blood, and tissues (Gotoh et al., 2024). A study by Lv et al. (2023) used neural stem/progenitor cells and their exosomes for treatment of cerebral palsy. Collectively, intranasal delivery was performed using repeated dosing schedules, varying from days to weeks, with follow-up periods ranging from 1 week to 24 months depending on the study type. The included studies followed interventional study designs, with the single-group design being the most used method, followed by parallel, sequential, and lesser usage of crossover and factorial designs (Table 2). Intranasal stem cell delivery was administered as a liquid suspension via nasal drops, drips, or sprays, whereas stem cell-derived exosomes were given as intranasal drops or MSC-exosome suspension sprays (Wang et al., 2023). Patients were positioned in a supine or reclined position to facilitate nose-to-brain transport (Li et al., 2018; Xie et al., 2023). Across all included studies, intranasal stem cells were reported to be safe, feasible, and well-tolerated (Lv et al., 2023; Valeri and Mazzon, 2023).

**Table 2:** Characteristics of included studies.

Registration number	Country	Method	Study design	Phase	Cell type	Cell dose	Disease condition	Transplant method	Study status	Primay outcomes	Secondary outcomes	Follow-up
NCT02795052 (Qiu et al., 2025)	USA, UAE (2016)	NEST Study - Single-arm group	18 Y and older (*n* = 500)	NA	Autologous BM-MSCs	NA	Nervous system disorders	IV and IN (lower 1/3 of nose)	Recruiting	Neuro-QOL SCALE, Activities of Daily Living (ADL)	NA	1,3,6 & 12 M
NCT03005249 (Lv et al., 2023)	China (2016)	RCT-Parellel group	3 to 12 Y; Treatment group-NSCs and rehabilitation therapy (*n* = 15) & The rehabilitation therapy control group (*n* = 10)	I/II	Allogeneic NSCs	5 × 10^5^/kg	Children with ischemic/ hypoxic CP 63.6% -spastic (quadriplegic). 31.8% - spastic dyskinetic 4.5% - spastic + ataxic	IN	Unknown	NSCs was well tolerated & GFFM-88 score improved in treatment group	Decrease in SDSC score; FFBN- ↓ed. brain network energy; VBM analysis- ↑ grey matter volume.	1,3,6,9 & 12 M
NCT04146519 (Boika et al., 2020)	Belarus (2017)	RCT-Parellel group	18 to 69 Y; Systemic (*n* = 6) & Tandem (*n* = 6)	II/III	Autologous BM-MSCs	Systemic IV- 0.5–2.0 million/kg & Tandem IN - 2–5 mL (5.0–12.6 million cells), followed by IV 10–50 million cells after 7 days.	Parkinson’s disease	Systemic (IV) & Tandem (IN + IV)	Unknown	Significant ↓ the severity of motor and nonmotor symptoms in the study group in the posttransplant period	Mood improvement, significant ↓ in daytime sleepiness in 1 M. decreased depressive mood in 3 M need long term follow up	1 & 3 M
NCT03724136	USA UAE (2018)	Non RCT-Parellel group	18 Y and older; (*n* = 100) Arm 1: IV BMSC fraction Arm 2: IV BMSC + near infrared light ARM 3: IV + IN BMSC	NA	Autologous BM-MSCs	IV – 14 cc IN – 1 cc in all arms	Alzheimer’s disease (with autism & dementia)	IV & IN (nasal mucosa topical)	Enrolling by invitation	MMSE and ASD assessed	ADL measured	1,3,6 & 12 M
NCT03899298	NA (2019)	Non-RCT-Parellel group	18 Y and older; (*n* = 5,000) IV infusion + nebulizer	I	Amniotic and umbilical Cord derived MSCs	NA	Neurological and pulmonary disease	IV & IN (nebulizer)	Not yet recruiting	DASH, SHIM, KDQOL, MMSE, COPD score measured	NA	1,3,8,12,18, 24,30,36 M and then on average each Y, up to 10 Y
NCT02192736	Panama (2020)	Single group	21 to 60 Y; Treatment group (*n* = 20)	I/II	Allogeneic mesenchymal trophic factors (MTF) from human (UC-MSC)	MTF once a w for a period of 4 w	Asthma	IN	Unknown	Safe and useful procedure for inducing improvements in pulmonary function and quality of life in patients with asthma.	Change in FEV1 and FVC measured	1,2,3 and 4 w & 1 M
NCT04388982 (Xie et al., 2023)	China (2020)	Non RCT – sequential group, pilot study with 3 + 3 design	50 to 80 Y; Treatment group: Low dose (*n* = 3) Medium dose (*n* = 3) High dose (*n* = 3)	I/II	Allogenic adipose tissue derived MSCs-Exos (ahaMSCs-Exos)	Low dose: 2 × 10^8^ particles in 1 mL saline. Medium dose: 4 × 10^8^ particles in 1 mL saline High dose: 8 × 10^8^ particles in 1 mL saline (two times per w through nasal spray devices)	Mild to moderate dementia due to alzheimer disease	IN (nasal drip)	Unknown	ADAS- cog, MMSE and MoCA- B scores suggests better cognitive function in medium dose arm	1 DLT in low and high dose and 0 DLT in medium dose,making middle-arm safer and well tolerated, and a dose of at least 4 × 10^8^ particles, 1 mL as safer dose ADCS- ADL shows improvementin second arm	16, 24, 36 and 48 w
NCT03356821 (Wagenaar et al., 2025)	Netherlands (2020)	open-label, single-center intervention study	Near term Neonates-1 to 10 D of age; Treatment group (*n* = 10)	I/II	Allogenic BM-MSCs	50×10^6^ BM-MSC	Perinatal Arterial Ischemic Stroke (PAIS) Neonatal stroke	IN	Completed	No adverse event noted in 9 babies within 24 h and 1 baby had fever has adverse effect	Long-term safety and tolerability were assessed at 3 M of age, and none of the babies showed adverse effects.	24 h & 3 M of age
NCT04602104 (Sánchez et al., 2025)	China (2020)	RCT-double blinded, parallel study	18 to 70 Y and older adults; (*n* = 18) Treatment group & Control group (placebo)	I/II	Allogeneic MSCs exosomes (hMSC-Exos)	Phase 1 Arm1: 2 × 10^8^ particles Arm2: 8 × 10^8^ particles Arm3: 16 × 10^8^ particles Phase 2 Arm 1: basic treatment+ hMSC-Exos (1/4 of MTD/day) Arm 2: basic treatment + hMSC-Exos (MTD/day) Arm 3: basic treatment + normal saline	ARDS	IN	Completed	To measure the murray lung injury score, PaO2/FiO2, SOFA score, ApachII score, number of days in ICU.	Incidence of adverse event, duration of clinical improvement,and 28 days mortality will be measured.	1, 2, 3, 4, 5, 6, 7, 14, 28 and 60 D
NCT05158101	Antigua and Barbuda, Argentina (2022)	Single group assignment	Children, younger and older adults; Treatment group (*n* = 15)	I	Allogenic umbilical cord derived exosomes	4 cc AlloEx (800 billion exosomes/day) for 2 days	Stroke	IN	Recruiting	To measure adverse events	NA	1, 6, 12, 24, 36, and 48 M
NCT05008588 (Nistor-Cseppentö et al., 2022)	Indonesia (2022)	RCT – factorial study	25 TO 60 Y; (*n* = 15) Arm 1 = conditioned medium + intra peritoneal transplantation of UC-MSCs Arm 2 = Intra-parenchymal transplantation of UC-MSCs Arm 3 = control group with neurotrophic drugs	I/II	UC-MSCs	Arm 1 = 3 CCs CM + 20×10^6^ UC-MSCs Arm 2 = 20×10^6^ UC-MSCs Arm 3 = neurologic and neurotrophic drugs	Ischemic stroke	IN conditioned medium + Intraparenchymal transplantation	Unknown	To measure changes in Brain-derived neutrophic factor, VEGF, MRI	To monitor progress through NIHSS and mRS score for evaluating neurological progress	1, 3, and 6 M
NCT05490173	Russia (2022)	RCT-Parellel group, Pilot experimental study	ELBW infant: 1 to 3 D of age born at gestational age 25/0-27/6 weeks; *N* = 10 Arm 1 = conditioned medium + intra peritoneal transplantation of UC-MSCs Arm 2 = Intra-parenchymal transplantation of UC-MSCs Arm 3 = control group with neurotrophic drugs	NA	Exosomes derived from MSCs	50 μL with an interval of 2-3 min (120 million MSCs) for 5 D	Premature birth Extreme prematurity Preterm intraventricular hemorrhage Hypoxia-ischemia, and Cerebral neurodevelopmental disorders	IN	Not yet recruiting	To measure the death incidence, survival with any of either severe IVH, PVL, or brain injury on cranial USG & MRI	To measue long term effect and feasibility	upto 40 w of age
NCT05152394	Antigua and Barbuda (2024)	Single group study	Child, adult and older adults; Treatment group (*n* = 20)	I	Allogeneic UC-MSC exosomes	4 CCs of AlloEx (800 million exosomes/day) for 2 days	Parkinson’s disease	IN	Recruiting	To measure safety and efficacy by monitoring adverse event and complication.	NA	1, 6, 12, 24, 36, and 48 M
NCT07105371 (Prodromos, 2025)	Antigua and Barbuda (2024)	Single arm non-controlled study	18 Y and older; Treatment group (*n* = 18) {ALS (*n* = 14) Kennedy disease (*n* = 1) Congenital myasthenic syndrome(*n* = 1) Lewy body dementia(*n* = 2)}	I	Allogeneic UC-MSC exosomes	32 treatments of AlloEx Exosome Group 1: single dose (*n* = 10) Group 2: 1.25 × single dose (*n* = 1) Group 3: 2 × single dose for 2 consecutive days (*n* = 8) Group 4: 2.25 × single dose for two consecutive days(*n* = 1) Group 5: total dose of 2.5 × AlloEx Exosomes for two consecutive D (*n* = 12)	Motor disorders ALS, Kennedy disease, Congenital myasthenic syndrome, Lewy body dementia	IN	Completed	No adverse events reported; Out of 18, 15 had near improvement, 2 had partial improvement and only 1 patient did not show significant response.	Repeat treatment generally showed incremental improvement above the improvement from prior treatment	1, 3, 6 M
NCT06598202 (Qiu et al., 2025)	Florida, US (2024)	multicenter, randomized, double-blind, placebo-controlled, dose-escalation trial	18 to 80 Y; Part 1: cohort 1,2,3 Part 2: Treatment group (*n* = 10) & Control group, exosomes placebo (*N* = 10)	I/II	Umbilical cord blood derived MSC exosomes (hUC-MSC-sEV-001)	1 mL per nostril, administered once daily, twice a w, for 2w	ALS	IN	Recruiting	DLT measured using CTCAE. To monitor ALSFRS-R, ALSAQ-40, ECAS, NI, CMAP and ROADS rating scale	To assess the feasibility by neuroimaging and blood markers (CRP, IL-6).	4 ± 1 w, 12 ± 1 w, 24 ± 1 w
NCT07232563	China (2025)	Single group study	18 to 70 Y; Treatment group (*n* = 9)	I	MSCs derived small extracellular vesicles of umbilical cord (hUC-MSC-sEV-001)	nasal drip for a total of 4 times, with a dosage of 1 × 10^11^articles per dose	Ischemic stroke	IN	Not yet recruiting	DLT measured via CTCAE, NIHSS and CT brain	NA	D-7 (+/-1), D- 14 (+/-2), and D-90 (+/-7) post-enrollment
NCT06858254	Boulder, USA (2025)	RCT- crossover study	40 to 75 Y; Treatment group (*n* = 60)	I/II	Autologous BM-MSCs	NA	Atypical parkinsonism Parkinson Disease Parkinson’s Plus Syndrome	Sham IV + INA BMAC 6 M IV BMA + Sham INA 12 M IV BMA + INA BMAC	Not yet recruiting	outcome measured by MDS-UPDRS, SPES/SCOPA, PSP-CDS to monitor motor function progress	NA	3, 6, 9, 12, 15 and 18 M
Morita et al. (2024)	Japan (2024)	Single group	(*n* = 18)	I	AD-MSC exosomes	3 vials (each containing 1 × 10^8^ exosomes) per week for 8 weeks.	Alzheimer’s Disease	IN exosomes	completed	HDS-R, blood pressure, pulse, blood biochemistry tests, measurements of hormones, and MCI screening test (9 protein types)	NA	baseline

Y, years; M, months; w, weeks; D, days; *n*, number of participants; N/A, not available; RCT, randomized controlled trial; IV, intravenous; IN, intranasal; MSC, mesenchymal stem cells; NSC, neural stem cell; UC-MSC, umbilical cord derived mesenchymal stem cells; BM-MSC, bone marrow derived mesenchymal stem cells; NEST study, neurologic stem cell treatment study; ADL, activities of daily living; Neuro-QOL, neurology quality of life; GMFM-88 score, gross motor function measure-88 score; SDSC, sleep disturbance scale for children; FBN, functional brain network; VBM, voxel-based morphometry; DASH, disabilities of arm, shoulder, hand questionnaire; SHIM, sexual health inventory for men questionnaire; KDQOL, kidney disease and quality of life questionnaire; AQOL, assessment of quality of life questionnaire; COPD, chronic obstructive pulmonary disease; MMSE, mini mental state examination; FEV_1_, forced expiratory volume for 1 s; FVC, forced vital capacity; ADAS-cog, Alzheimer ‘s disease assessment scale-cognitive section; ADCS-ADL, Alzheimer’s disease cooperative study activities of daily living; MoCA-B, montreal cognitive assessment-basic; DLT, dose limiting toxicity; PaO_2_/FiO_2_, the ratio of alveolar oxygen partial pressure to fraction of inspired oxygen; SOFA Score, sequential organ failure assessment score; APACHE II, acute physiology and chronic health evaluation II; NIHSS, National Institutes of Health Stroke Scale; mRS, modified Rankin scale; MRI, magnetic resonance imaging; ELBW, extremely low birth weight; IVH, intraventricular hemorrhage; PVL, cystic periventricular leukomalacia; USG, ultrasound; CTCAE, common terminology criteria for adverse events version; ALSFRS-R, ALS functional rating scale-revised; ALSAQ-40, amyotrophic lateral sclerosis assessment questionnaire; ECAS, edinburgh cognitive and behavioral als screen; NI, neurophysiological index; CMAP, compound muscle action potential score; ROADS, Rasch overall ALS disability scale; CRP, C reactive protein; IL-6, interleukin 6; ALSFRS-R, amyotrophic lateral sclerosis functional rating scale; CT, computed tomography; INABMAC, intranasal bone marrow aspirate administration; IV BMA, intravenous bone marrow aspirate administration; MDS-UPDRS, movement-disorder society unified parkinson disease rating scale; SPES, short parkinson’s evaluation scale; SCOPA, scales for outcomes in Parkinson’s disease-motor function; PSP-CDS, progressive supranuclear palsy clinical deficits scale.

No serious adverse effects were reported. Several studies reported improvement in motor and cognitive functions. Safety was assessed by monitoring adverse effects following intranasal administration; follow-up duration differed based on the trial. Some studies used MRI to evaluate the occurrence of infection and cerebral tumorigenicity on long-term follow-up (Li Y. et al., 2022; Valeri and Mazzon, 2023). However, outcome measures varied, and most studies lacked definitive conclusions regarding efficacy.

### 3.4 Risk of bias assessment

The risk of bias of the 19 included studies was assessed using the Cochrane Risk of Bias 2 (RoB 2) tool, supplemented by the Cochrane guidelines for registry-only and grey literature records. No study achieved an overall low-risk judgment. One published article (Lv et al., 2023) was rated as having ‘some concerns’ overall, representing the highest methodological quality in the pool. The remaining six published articles and all 12 registry or grey literature records were rated as ‘high risk’ (*n* = 14 overall high risk, 74%) or ‘some concerns’ for registry records with favorable design features (*n* = 5, 26%).

**Figure 2:**
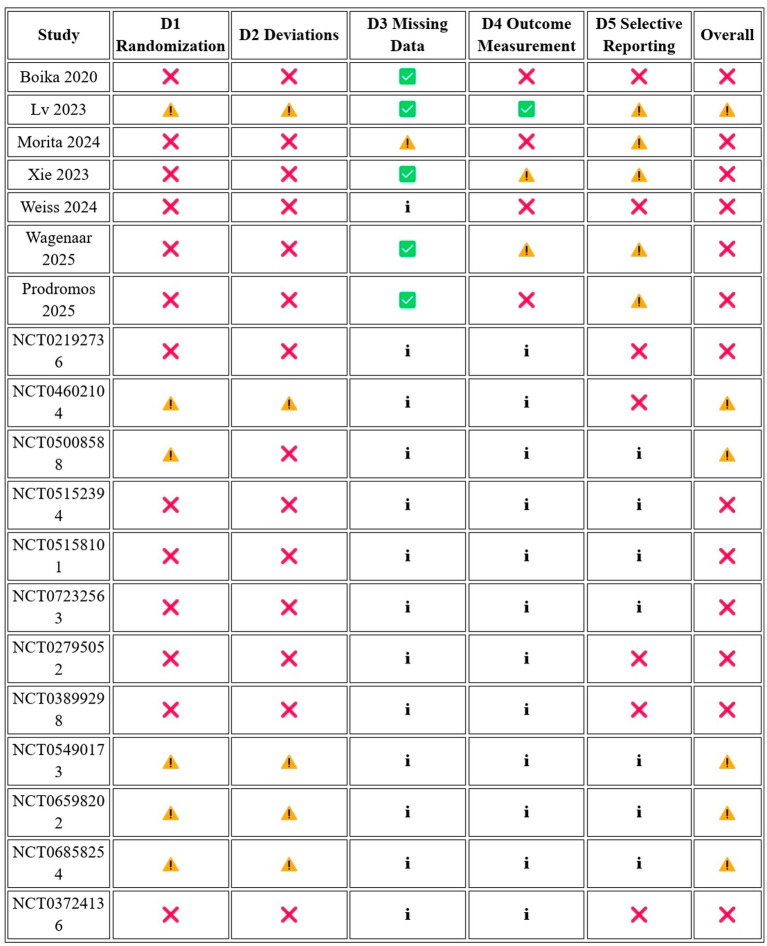
Summary of Risk of Bias 2.

Across the published studies, the most consistently compromised domains were Domain 1 (randomization: 0% low risk) and Domain 2 (deviations from intervention: 0% low risk), reflecting the absence of formal randomization procedures and the open-label design without sham or placebo control in all published studies. Domain 4 (outcome measurement) was rated low risk in only two published studies, Lv et al. (2023), which explicitly reported blinded outcome assessors, and partially in Xie et al. (2023), where objective PET-MRI biomarker outcomes mitigated some detection bias concern. Domain 5 (selective reporting) was rated high risk in majority of the studies given the absence of a registered protocol for the systematic review itself, as well as the absence of posted results from multiple long-running or completed trials (Figure 2).

**Figure 3:**
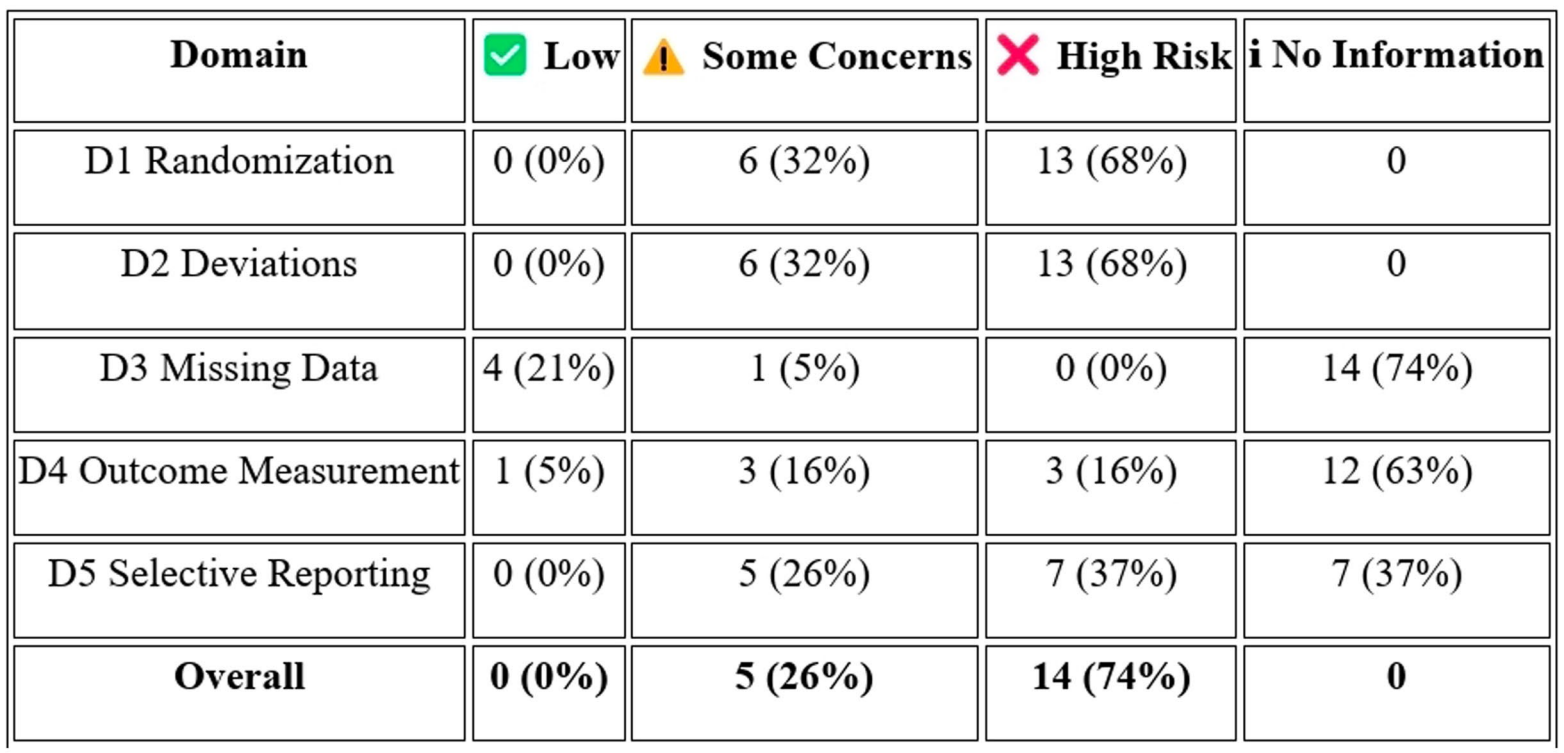
Risk of bias domain-level summary (*n* = 19).

Among the registry-only records, Domains 3 and 4 were mandatorily rated as ‘no information’ per Cochrane guidance, as no published results were available for assessment. Five registry-only trials demonstrated design features meriting a judgment of some concerns rather than high risk, specifically their use of randomized allocation (NCT04602104, NCT05008588, NCT05490173, NCT06598202, NCT06858254), with two additionally specifying multi-level blinding protocols (NCT06598202: quadruple-blind; NCT06858254: participant-blinded crossover with sham procedure). All efficacy estimates are drawn exclusively from high-risk or some-concerns studies, and no published controlled trial with adequate randomization and blinding exists in this field at this time (Figure 3).

### 3.5 Publication bias

**Figure 4:**
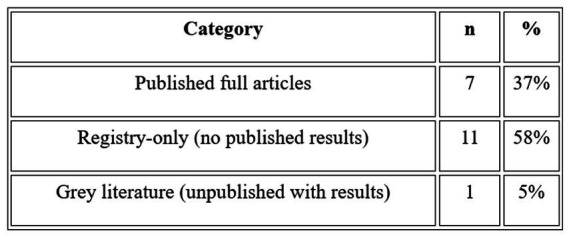
Structural publication assessment.

Structural publication bias is a significant concern in this systematic review. Of the 19 included records, 11 are registry-only entries (58%) and one is unpublished grey literature (5%), meaning that only 37% of the identified evidence base consists of peer-reviewed published articles, substantially exceeding the 30% threshold at which structural publication bias is considered a major threat to review validity (Figure 4). Particularly concerning is that at least 5 studies with substantial registration histories show patterns consistent with non-publication of results. NCT04602104, a completed randomized double-blind placebo-controlled Phase 2 trial with actual enrolment of 18 participants (completed January 2023), has posted no results more than 2 years after completion. NCT02192736, last recorded as active-not-recruiting in 2018 with an estimated primary completion date of April 2020, has posted no results after more than 5 years. NCT03724136 and NCT02795052, both enrolling patients since 2018 and 2016, respectively, with estimated cumulative enrolments of up to 600 participants, have yielded no systematic published results after over 6 and 9 years of operation respectively, with only a single-patient conference poster representing the public output of the former. Two further studies (NCT03899298) appear to have been registered but never operationalized. This pattern of trial registration without results dissemination is consistent with selective non-reporting of neutral or negative findings, as identified in other fields of regenerative medicine. As the number of published studies with extractable quantitative effect sizes is fewer than 10, contour-enhanced funnel plot analysis and Egger’s regression test are not applicable at this time. All efficacy conclusions in this review should be interpreted in the context of likely favorable outcome publication bias.

### 3.6 ROBINS-I assessment

**Figure 5:**
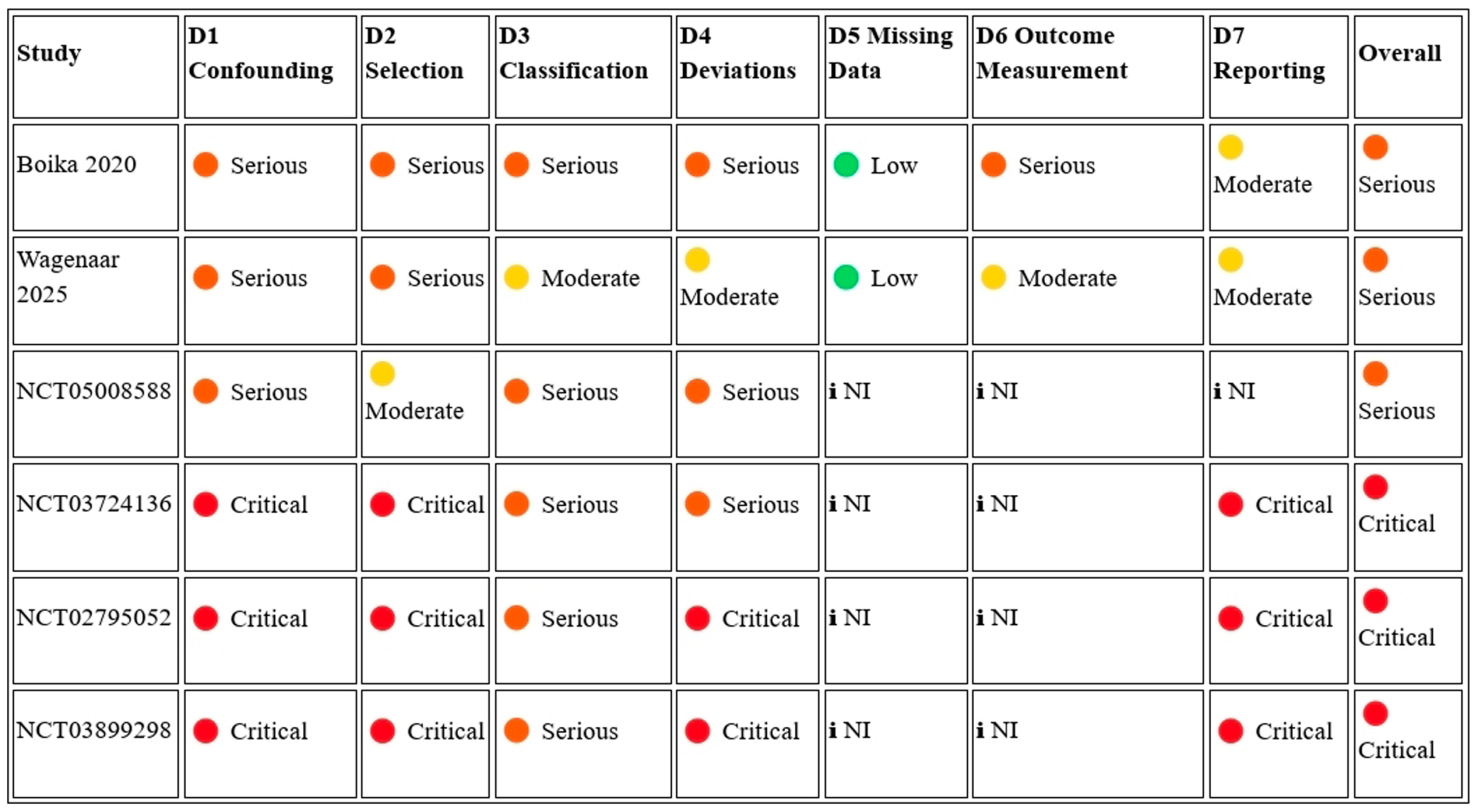
ROBINS-I aggregation summary.

Six non-randomized comparative studies were assessed using the Risk of Bias in Non-randomized Studies of Interventions (ROBINS-I) tool (Figure 5). Two studies (NCT03724136 and NCT02795052) were rated critical overall, and a third (NCT03899298) was also rated critical given its apparent non-operationalization after registration. The remaining three studies (Boika et al., 2020, Wagenaar et al., 2025, and NCT05008588) were rated serious overall. No non-randomized study in this review achieved an overall low or moderate risk of bias rating. The most pervasive bias domain was confounding (Domain 1), which was rated critical in three studies due to the absence of any concurrent control group with comparisons made only against published literature benchmarks or by-invitation-only cohorts. It was serious in the 3 remaining studies due to unbalanced baseline characteristics, confounding by indication, or the inseparability of intranasal from concurrent invasive co-interventions. Selection bias (Domain 2) was rated critical in 4 studies, reflecting self-selected patient-funded populations, by-invitation enrolment, or subjective prognostic eligibility criteria. Selective outcome reporting (Domain 7) was rated critical in 3 registry-based studies that have been enrolling patients for 6 to 9 years without posting systematic results — a pattern consistent with critical publication bias. Only 2 studies achieved low risk on any individual domain. Both Boika et al. (2020) and Wagenaar et al. (2025) had low risk for missing data (Domain 5), reflecting complete outcome ascertainment in their respective study populations. No non-randomized study in this pool can support reliable causal inferences about the efficacy of intranasal stem cell administration, and any efficacy findings from these studies should be treated as exploratory hypothesis-generating evidence only (Kiarashi et al., 2024; Li M. et al., 2022; Deokate et al., 2024; Demirezen Alper, 2024; Guo et al., 2021; Allouh et al., 2025; Sajjad et al., 2024)

## 4 Discussion

### 4.1 Mechanism of action

**Figure 6:**
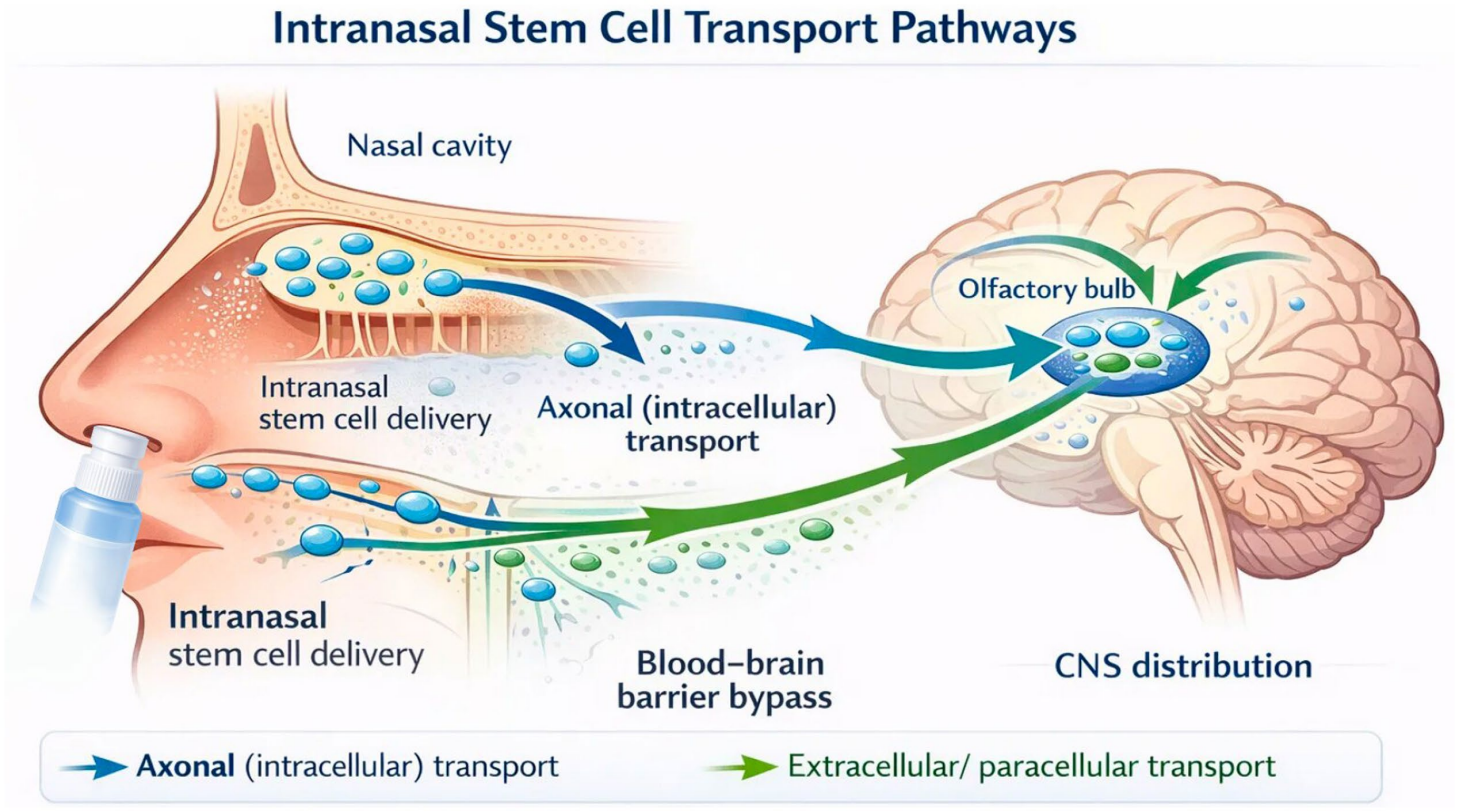
Schematic representation of intranasal stem cell delivery showing axonal (intracellular) and extracellular/paracellular pathways from the nasal cavity to the olfactory bulb and CNS distribution.

Intranasal stem cell administration is an emerging approach to transplantation (Figure 6). It is a nose-to-brain delivery system that delivers stem cells and their derivatives directly into the CNS by bypassing the BBB. The therapeutic effects of stem cells are governed by paracrine signaling, neuroprotection, and immunomodulation rather than direct replacement (Guo et al., 2021; Wu et al., 2025).

### 4.2 Paracrine mechanism

**Figure 7:**
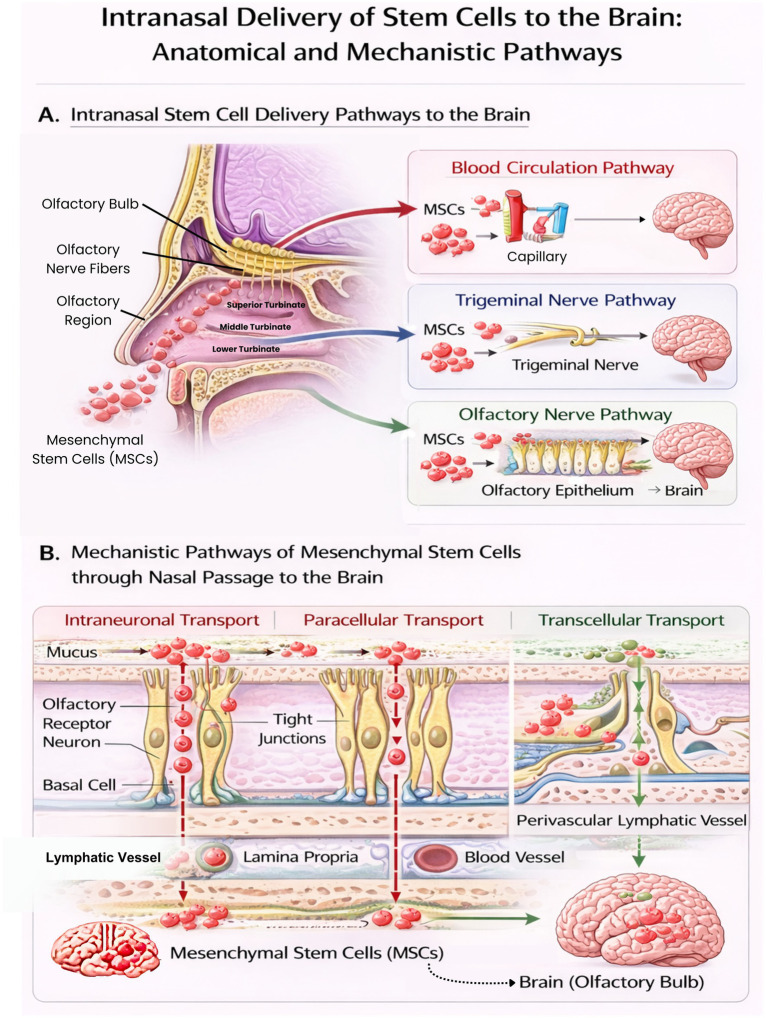
Intranasal stem cells delivery pathways to the central nervous system. Schematic representation of intranasal administration of how the stem cells are transported across the nasal mucosa and the olfactory epithelium. The therapeutic agents enter the brain through neuronal, paracellular and transcellular routes with the olfactory and trigeminal nerve routes, and supplementary systemic vascular and lymphatic absorption.

MSCs exhibit therapeutic effects through paracrine signaling rather than direct differentiation (Figure 7). MSCs decrease the concentration of reactive oxygen species (ROS) and inflammatory cytokines to suppress the activation of neuroglial cells and promote the regeneration of vessels and neurons. After intranasal administration, upon reaching the nasal mucosa, MSCs and their derivatives secrete bioactive substances, including extracellular vesicles, inflammatory cytokines, chemokines, growth factors, and extracellular matrix (ECM) components. These substances are released by exocytosis, fusing with the plasma membrane and regulating the surrounding tissue environment.

Exosomes facilitate cell-to-cell communication by passing through the BBB and transferring proteins, lipids, and microRNAs for therapeutic effects, including promoting angiogenesis, enabling tissue remodeling, and decreasing apoptosis (Han et al., 2022). Chemokines and their receptors (CXCL9, CXCL16, CCL20, CCL25) maintain MSC homing. Inflammatory cytokines, such as TNF-*α* and IFN-*γ*, increase the migratory capacity of MSCs (Sajjad et al., 2024). MSCs produce CX3CL1 chemokine to target CX3CR1 on microglia, controlling their activation and phagocytosis (Han et al., 2022).

Growth factors secreted by MSCs include vascular endothelial growth factor (VEGF), keratinocyte growth factor (KGF), basic fibroblast growth factor (bFGF), insulin-like growth factors (IGF-1 and IGF-2), and hepatocyte growth factor (HGF). These factors are responsible for tissue differentiation, tissue repair, and suppression of fibrogenesis. MSC homing follows five steps: rolling (CD44), chemokine activation (CXCR4), firm arrest (integrin), diapedesis (MMPs), and finally migration to the injured site by recognizing the chemotactic signal (Sajjad et al., 2024).

### 4.3 Neuroprotection

MSCs and their derivatives reach the target site through paracrine signaling, passing through the branches of the trigeminal and olfactory pathways and releasing neuroprotective and anti-apoptotic factors. The pleiotropic properties of MSCs include anti-apoptosis, angiogenesis, growth factor production, neuroprotection, anti-fibrosis, and chemo-attraction. These properties have the capacity to suppress inflammation and reduce the pathogenic immune response (Li M. et al., 2022). MSCs promote neurovascular remodeling and angiogenesis through secretion of VEGF and restore blood flow in the brain after ischaemia or infarct (Zhang et al., 2020). MSC-derived exosomes are now widely used in clinical trials to monitor neuroprotective and immunomodulatory effects (Fan et al., 2020).

### 4.4 Immunomodulation

MSCs maintain immunoregulatory functions and tissue repair by balancing innate and adaptive immune cells. MSCs suppress inflammatory responses by inhibiting T cell, B cell, and NK cell proliferation and by reducing cytokine release (Alvites et al., 2022). Cytokines promote proliferation of regulatory T cells (Tregs), which suppress killer T cells that attack foreign cells or tissues. These characteristics make MSCs safer for allogeneic transplantation (Yang et al., 2023). MSCs possess low immunogenicity because they have low expression of MHC class I complex, and co-stimulatory molecules remain unexpressed (Dabrowska et al., 2021).

The therapeutic effects of NSCs and MSC-derived exosomes are manifested in distinct pathways in the included studies. NSCs have been reported to have the ability to form neurons and glial cells. This directly contributes to neuronal repair, glial differentiation and regeneration, the release of neurotrophic factors, and immune regulation, thereby facilitating neuronal survival and endogenous recovery processes (L. Yang et al., 2024). MSC-derived exosomes serve as a cell-free delivery vehicle that can transport bioactive molecules, such as proteins, RNAs, and microRNAs, to the target site within the CNS. Their smaller size (40-200 nm), enables efficient intranasal delivery of exosomes. They bypass the BBB through olfactory pathways and reach deep brain regions quickly- typically in less than 30 min. This reportedly allows exosomes to help in processes involving neuroinflammation, apoptosis, angiogenesis, and immunomodulation (Gotoh et al., 2024; Waseem et al., 2023; Fan et al., 2020; Han et al., 2022; Boika et al., 2020; Prodromos, 2025; Salehi et al., 2022; Salehi et al., 2022; Guo et al., 2021; Li Y. et al., 2022; Zhang et al., 2023; Park and Riew, 2025; Zhang et al., 2023; Crowe and Hsu, 2022; Park and Riew, 2025; Crowe and Hsu, 2022; Park and Riew, 2025; Zhang et al., 2021).

### 4.5 Methods of intranasal stem cell delivery

Various methods have been described for intranasal stem cell delivery. Table 3 summarizes the main approaches, including manual nasal drops (Alvites et al., 2022), nasal patch-assisted infusion (Lv et al., 2023), delivery of small extracellular vesicles (Crowe and Hsu, 2022), and advanced intranasal devices such as nebulizers and bi-directional sprays (Shen et al., 2023). Each method presents distinct advantages and limitations regarding dosing precision, patient compliance, BBB bypassing capability, and clinical evidence (Platt et al., 2020; Zhang et al., 2023; Qiu et al., 2025; Prodromos, 2025; Isik et al., 2023; Qiu et al., 2025; Rahimi Darehbagh et al., 2024; Ru et al., 2025; Yang, 2025; Sánchez et al., 2025; Alvites et al., 2022; Li Y. et al., 2022; Xie et al., 2022; Lv et al., 2023; Guo et al., 2021; Sajjad et al., 2024).

**Table 3:** Comparison of methods for administering stem cells and their derivatives intranasally.

Method/Device	Brief description	Advantages	Limitations	References
Manual nasal drop of cell suspension	Stem cells or exosomes in liquid form are administered into each nostril with syringe. Patient in supine or semi-reclined position.	Non-invasive, repeated dosing is possible, bypasses BBB, retention time 14 min, surgery not needed & pediatric friendly.	Variable dosing to olfactory region. Limited volume. Not self-administrable.	Lv et al. (2023), Zhang et al. (2020), and Zhang et al. (2023)
Nasal patch–assisted intranasal infusion (CP trial)	NSC suspension applied intranasally and covered with absorbent nasal patches.	Prolonged contact time with nasal mucosa. Simple to administer.	Nasal patch detachment. No real time dose control.	Crowe and Hsu (2022), Lv et al. (2023), and Zhang et al. (2020)
Intranasal delivery of stem-cell-derived small extracellular vesicles (Exosomes)	Concentrated EVs/exosomes dripped intranasally in small volumes.	Nano carriers bypass BBB. Reaches CNS via perivascular pathways.	Mucociliary clearance under study.	Crowe and Hsu (2022) and Shen et al. (2023)
Advanced intranasal devices (Nebulizers, bi-directional sprays, atomizer)	Stem cells can be delivered by nebulizer and bi-directional sprays.	Targets on upper nasal cavity, direct CNS targeting. Repeated dosing.	Human data not yet reported. Can be trapped in lung.	Shen et al. (2023)

## 5 Pharmacokinetics and pharmacodynamics considerations

The route of administration directly influences the pharmacokinetics and dynamics of administered stem cells (Table 4). According to Li M. et al. (2022) the bioavailability of an intranasally administered drug is approximately 10 times higher than systemic administration. This route is favorable for its rapid onset of action. Generally, within brain regions, the concentration of intranasally administered drugs is directly proportional to the concentration reaching the brain tissue, making the intranasal route a more reliable and efficient method compared to systemic injections. Being non-invasive, it can support frequent dosing (Drath et al., 2025; Li Y. et al., 2022).

**Table 4:** Comparison of intranasal delivery with other common routes for CNS targeting.

Characteristics	Intranasal	Intravenous	Intrathecal	References
Administration site	Nasal mucosa	Blood stream	CSF	Lv et al. (2023)
Invasiveness	Non-invasive	Less Invasive (Vein puncture)	Invasive (Lumbar puncture)	Owolabi et al. (2023) and Vargas-Rodríguez et al. (2023)
First pass metabolism	No	Yes	No	Zhang et al. (2020)
CNS targeting	Direct	Indirect (pulmonary, liver, and Spleen entrapment)	Direct	Zhang et al. (2020)
Cells reaching brain	Large volume	Only Small Volume	Large volume but decrease in stem cells after few days	Guo et al. (2021) and Li Y. et al. (2022)
BBB bypassing	Yes	No	Yes	Lv et al. (2023) and Zhang et al. (2021)
Possibility of Repeated dosing	Must	May be	Very rare	Valeri and Mazzon (2023)
Disadvantages	Lack of clinical trials.	Pulmonary, liver, spleen entrapment. Risk of PE.	Blockage of CSF flow due to MSC aggregation.	Guo et al. (2021), Valeri and Mazzon (2023), and Zhang et al. (2020)

A study by Li Y. et al. (2022) compared the duration for stem cells and their derivatives to reach the brain in preclinical studies. Results varied from 15 min to a maximum of 4 months across various disease models. Stem cells were found in all regions of the brain and spinal cord. In PD model mice, stem cells were distributed throughout the brain within 7 days after intranasal administration, with approximately 20% found in the brainstem and olfactory bulb. Overall, cells survived at the target for prolonged durations (7 days to 4 months), suggesting that the intranasal route has a good retention capacity (Li Y. et al., 2022). However, pharmacokinetics data from clinical trials remain limited.

The pharmacodynamics of preclinical studies showed improvement in cognitive function, behavior, and memory, while reducing inflammation (Li Y. et al., 2022).

Clinical trial data on PD patients showed improvement in mood, significant decrease in daytime sleepiness within one month, and decreased depressive mood within 3 months (Boika et al., 2020). In another study, treatment with medium-dose exosomes showed significant improvement in cognitive function, suggesting this dose as safer for mild to moderate Alzheimer’s dementia (Li Y. et al., 2022; Xie et al., 2023).

The long-term safety and tolerability of MSCs were demonstrated in the PAIS study, where BM-MSCs were administered safely without significant side effects (Valeri and Mazzon, 2023). Allogenic umbilical cord-derived exosomes also showed significant improvement in motor function in patients with ALS and motor diseases, with preliminary evidence for repeated dosing showing incremental improvement (Prodromos, 2025). Pharmacodynamics can be evaluated using diagnostic imaging techniques including fluorescence microscopy, MRI, and SPECT scans, which track the migration of intranasally administered stem cells (Hong et al., 2023; Li et al., 2018). A study by Li et al. (2018) suggests that SPECT scan may serve as a more reliable imaging tool than MRI, as MRI can show false positives due to iron oxide components (Li et al., 2018).

### 5.1 Limitations

The included studies were characterized by pervasive and multidimensional methodological limitations that collectively prevented reliable causal inference about the safety and efficacy of intranasal stem cell and derivative therapies for neurological and respiratory diseases.

Under RoB 2 assessment, not a single study across all 19 included records achieved low risk in Domain 1 (randomization) or Domain 2 (deviations from intervention). No published trial in this field has implemented adequate allocation concealment, and no published comparative study has employed a sham or placebo intranasal instillation as a control. The single study rated as having overall some concerns represented the strongest design in the pool precisely because it reported blinded outcome assessors. However, even this study lacked a sham intranasal procedure in the control arm (Lv et al., 2023). This absence of blinding is particularly consequential for outcome measure scales, which can be substantially susceptible to expectation and detection bias in open-label designs. The placebo response rate in Parkinson’s disease trials alone has been estimated at 16–40% on motor scales, meaning unblinded motor outcome data from this field cannot be interpreted at face value.

Under ROBINS-I assessment, 3 of 6 non-randomized comparative studies were rated critical for confounding. This was mainly due to the systematic differences between self-selected patient-funded treatment-seeking populations and the historical or literature-based comparators against which they were evaluated. Patients who seek out, travel internationally to access, and pay out-of-pocket for experimental cell therapy are systematically different from those represented in natural history cohorts or pharmacological trial comparators across multiple unmeasured dimensions. These include health literacy, socioeconomic status, motivation, functional reserve, and caregiver support. All of which independently predict better outcomes on Quality of life and functional assessments. Regression to the mean constitutes an additional and unquantifiable confound in studies enrolling patients at a point of functional plateau or recent clinical deterioration, generating apparent improvement on repeated measurement that is entirely independent of any treatment effect.

A fundamental challenge across this review’s study pool is the inconsistent classification of the intranasal intervention itself. Multiple studies combine intranasal and intravenous administration in the same patient (Boika et al., 2020; NCT05008588; NCT02795052; NCT03724136; NCT06858254), making it impossible to attribute any observed outcome to the intranasal delivery route specifically. Among the remaining studies, the biological products administered span five mechanistically distinct categories. These include MSCs, neural stem cells derived from fetal forebrain tissue, conditioned medium and secretome, purified exosome or extracellular vesicle preparations, and bone marrow aspirate fractions. The doses ranged from 5 × 10^5^ cells per kilogram to approximately 800 billion exosome particles. The treatment durations ranged from 2 days to 12 weeks. Furthermore, two studies targeting lower nasal passages for trigeminal pathway delivery (NCT02795052; NCT03724136) are mechanistically distinct from studies targeting the olfactory fissure for direct olfactory pathway to the brain (Lv et al., 2023; Wagenaar et al., 2025; Xie et al., 2023). However, both approaches were classified under the intervention label of intranasal administration. This degree of biological, dosimetric, and anatomical heterogeneity within the intervention category limits the pooled efficacy estimates of clinical interpretability.

The individual study samples ranged from 1 to 25 participants. None of the published studies reported a formal sample size calculation for efficacy endpoints, and none were powered to detect clinically meaningful treatment differences with adequate statistical confidence. This creates a high risk of both false-negative findings (insufficient power to detect real effects) and false-positive findings (overestimation of effect sizes in small samples). The median sample size of 13 analyzable participants per published study is insufficient for any primary efficacy conclusion regardless of the magnitude of observed differences.

This systematic review was not registered in PROSPERO or any equivalent prospective registry prior to the conduct of study selection, data extraction, and RoB 2 assessment. The absence of pre-registration means that decisions about inclusion criteria, outcome prioritization, subgroup analyses, and statistical models cannot be verified as independent of knowledge of the available data, introducing potential outcome-reporting and analytical-flexibility bias at the review level.

Two investigator clusters contributed multiple related studies to this review under conditions that raise material conflict of interest concerns. First, three studies from the Foundation for Orthopaedics and Regenerative Medicine (NCT05152394, NCT05158101, Prodromos, 2025) were conducted by the same principal investigator who served as the sole outcome assessor for the primary efficacy measure in the published study, under a patient-funded model in which participants paid for investigational treatment. Second, four co-authors of Xie et al. (2023) are current employees of the exosome manufacturer whose product was under evaluation. These conflicts do not invalidate the data from these studies, but they introduce material risk that observed findings may systematically overestimate benefit, and they must be explicitly considered when interpreting efficacy claims from these sources.

### 5.2 Evidence gaps

The RoB 2 and ROBINS-I assessments conducted in this review do not merely catalog methodological shortcomings in individual studies, they reveal a coherent and reproducible pattern of structural deficiencies that are specific to intranasal stem cell delivery trials as a field. The gaps identified below are not incidental to the studies assessed; they are the direct mechanistic consequences of the bias patterns found.

The field has not tested whether intranasal delivery works, only whether receiving it in an open-label setting produces measurable change. The most fundamental finding of this review’s bias assessment is that every published comparative study used an unblinded design without a sham intranasal control. This is not a minor design limitation. It is a categorical evidentiary failure that renders every published efficacy estimate in this field uninterpretable as evidence of biological activity. Domain 2 of RoB 2 was rated high risk across all 7 published studies and serious or critical in all 6 n-RCTs under ROBINS-I, due to the lack of a control group that underwent the same nasal procedure as the treated participants. As a result, the outcomes reported across all published studies with improvements in MDS-UPDRS, ALSFRS-R, ADAS-Cog, GMFM-88, and HDS-R represent the combined and inseparable effects of biological activity, procedural stimulation of the nasal mucosa and olfactory epithelium, expectation and placebo responses, and natural disease fluctuation. The intranasal route is particularly susceptible to this conflation because nasal manipulation, olfactory stimulation, and the sensory experience of administration are themselves potentially neuroactive. No existing published study can distinguish these components. This is the single most consequential gap in the field, and it must be addressed before any efficacy claim from an intranasal stem cell trial can be taken seriously.

The universal absence of assessor blinding means the field’s efficacy data are systematically biased toward positive findings. Domain 4 (outcome measurement) was rated low risk in only one published study (Lv et al., 2023) and high risk or some concerns in all others, with the consistent driver being unblinded outcome assessment on clinician-rated or patient-reported scales. The placebo response on motor scales in a PD study was estimated at 16–40% (Goetz et al., 2008). In Alzheimer’s disease trials, caregiver-reported outcomes are known to be substantially inflated by expectation in open-label settings (Schulz et al., 2013). Critically, in Prodromos (2025), the principal investigator served simultaneously as treating physician, hypothesis proponent, and sole outcome assessor for the primary efficacy measure. The systematic absence of assessor blinding across published studies means that the available data are structurally predisposed to overestimate benefit, and the magnitude of this overestimation cannot be quantified or corrected retrospectively. Every future trial in this field should treat independent blinded outcome assessment as a non-negotiable minimum standard and not an optional design refinement.

The n-RCTs are confounded by precisely the biases most likely to generate false positive signals. Under ROBINS-I, confounding was rated serious in three studies and critical in three others. The critical ratings were driven not by random failure of randomization but by systematic design choices. These include patient-funded enrollment models, by-invitation selection, subjective prognostic eligibility criteria, and comparison against literature benchmarks that together would affect the selection of patients most likely to improve, with a motivation to report improvement, and likely different from randomly selected comparator population.

The NEST (NCT02795052) and ACIST (NCT03724136) studies, which together represent the largest registered patient populations in this review (estimated *n* = 600), both operate under models where patients pay to participate in experimental cell therapy, are selected by subjective investigator judgment of their ‘potential for improvement,’ and are evaluated against historical or literature comparators rather than concurrent controls. These design features do not merely introduce bias. They structurally guarantee the appearance of benefit even in the complete absence of biological activity. That neither study has published systematic results after 6–9 years of operation is itself a critical finding: it suggests either that the findings are neutral or negative and have been selectively withheld, or that the data quality is insufficient for peer-reviewed publication both of which are deeply concerning for a field seeking clinical translation.

The absence of human pharmacokinetic and biodistribution data means dose selection across all trials has been arbitrary. No included study, published or registry-only reported quantitative pharmacokinetic or biodistribution data for intranasally administered cells or derivatives in human participants. All dose selection in published trials was extrapolated from rodent models or determined empirically through dose escalation without pharmacological justification. This gap is directly consequential for the interpretation of null or negative findings: it is currently impossible to determine whether a given dose reached the target brain region at a pharmacologically relevant concentration. Under Domain 1 of RoB 2, the inability to confirm target engagement compounds the already serious confounding concerns, a negative finding in an underdosed trial is uninformative, and a positive finding in a trial with unknown brain distribution cannot be mechanistically attributed to the intended delivery pathway. Human biodistribution studies using radiolabelled or otherwise trackable preparations should be embedded as mandatory primary endpoints in the next generation of Phase 1 trials.

The field’s publication bias has specifically prevented the accumulation of null and negative evidence. Sixty-three percent of identified studies are registry-only records with no published results, substantially exceeding the 30 % threshold at which structural publication bias is considered a critical threat to evidence synthesis. More specifically, the studies that have been enrolled the longest, with the largest estimated sample sizes, are precisely those that have produced no systematic published output including a completed double-blind placebo-controlled ARDS trial (NCT04602104, actual enrollment *n* = 18, completed January 2023, no results after 2 years), a six-year Alzheimer’s and cognitive impairment study (NCT03724136, estimated *n* = 100, zero results), and a nine-year broad neurological cohort (NCT02795052, estimated *n* = 500, zero results). The consequence is that the published literature on intranasal stem cell therapy consists almost entirely of small open-label studies reporting positive or inconclusive results, while the larger and potentially more rigorous studies remain systematically unpublished. This creates a published evidence base that is structurally skewed toward the appearance of benefit, meaning that any meta-analytic pooling of published data will systematically overestimate efficacy. This problem cannot be corrected by statistical methods in the absence of the missing data.

The field has never defined what a clinically meaningful response looks like for intranasal delivery specifically. No included study pre-specified a minimum clinically important difference (MCID) for any outcome measure as a threshold for defining treatment success. Under Domain 5 of RoB 2, the absence of pre-registration or pre-specified analysis plans was rated high risk in the majority of studies. This meant that analyses are conducted on accumulated data without pre-commitment to effect-size thresholds. Without pre-specified MCIDs, statistically significant improvements of uncertain clinical relevance, such as a 9% reduction in off-period MDS-UPDRS III (Boika et al., 2020) or a 1.9-point HDS-R improvement in Alzheimer’s patients with mean baseline scores of 15.6 (Morita et al., 2024) cannot be distinguished from clinically trivial variation in score, and the field has no shared standard against which to benchmark progress.

## 6 Conclusion

This systematic review set out to evaluate the human clinical evidence for intranasal administration of stem cells and their derivatives in neurological and respiratory diseases. The answer that emerges from a comprehensive bias assessment of 19 studies using RoB 2 and ROBINS-I is unambiguous, and it is important to state it plainly: the field does not yet have a single published clinical trial that can reliably answer whether intranasal stem cell or derivative therapy works in any human condition. This is not a judgment about the biological plausibility of the approach. The preclinical evidence for neurotropic, immunomodulatory, and regenerative effects of intranasally delivered MSC derivatives is substantial and growing. It is a judgment about what the existing human clinical evidence actually demonstrates, and what the universal pattern of bias found across all 19 studies reveals about where the field has systematically failed to build the evidentiary foundation that translation requires.

The bias pattern identified in this review is not random. It is coherent, directional, and field-wide. Across every published study, the same set of design failures recur. This includes open-label administration without sham intranasal control, unblinded outcome assessment on clinician-rated or patient-reported scales, absence of pre-registration or pre-specified analysis plans, and sample sizes too small to detect anything but large effects with statistical confidence. These are not isolated failures of individual investigators. They are collective failures of a field that has moved into human trials without establishing the methodological infrastructure that Phase 2 and Phase 3 evidence requires. The result is a published literature composed almost entirely of single-arm or non-randomized open-label studies whose efficacy estimates cannot be attributed to the biological activity of the intervention. Where treatment results cannot be distinguished from placebo and expectation effects, and cannot be replicated or refuted. As the conditions for meaningful replication blinded assessment, concurrent sham-controlled comparators, pre-specified endpoints have never been established.

The most revealing finding of this review is not any individual study’s result. It is what the pattern of bias, combined with the pattern of non-publication, collectively discloses about the field’s evidentiary state. Three of the 6 n-RCTs assessed under ROBINS-I received a critical overall rating. Not because of a single methodological failure but because of the simultaneous accumulation of critical confounding through self-selected patient-funded populations, critical selection bias through by-invitation enrollment with subjective prognostic eligibility, and critical selective reporting through years-long non-publication of systematic results from trials that have enrolled hundreds of patients. The NEST (NCT02795052) and ACIST (NCT03724136) studies between them represent an estimated 600 enrolled participants across nearly a decade of clinical operation, with zero peer-reviewed publications of systematic trial data. This is not a gap in the evidence. It is a structural failure of the field’s commitment to transparent scientific reporting, and it means that the published evidence base for intranasal stem cell therapy is not merely incomplete but is also perhaps systematically skewed toward the appearance of benefit. Any summary of the current evidence that does not foreground this fact risks misleading readers about the state of the field.

What the bias assessment of this review most clearly identifies is a specific and actionable catalog of what intranasal delivery trials are structurally missing. They are missing sham controls, inert intranasal instillation of vehicle solution, administered through the same procedure by the same personnel, in a setting indistinguishable from active treatment. They are missing blinded outcome assessors, independent evaluators with no knowledge of treatment allocation, assessing functional outcomes using standardized, validated instruments under conditions that preclude identification of group membership. They are missing pre-registered analysis plans, publicly committed, timestamped specifications of primary endpoints, MCIDs, statistical models, and subgroup analyses, made before data collection begins.

They are missing human pharmacokinetic and biodistribution data, evidence that the administered product reaches the target brain region at a concentration relevant to the proposed mechanism of action. They lack long-term safety surveillance and systematic monitoring of tumorigenicity, immune sensitization, and cumulative toxicity beyond the 3–12-month follow-up windows characteristic of published studies. And they are missing published results from completed trials, from long-running enrollment programs, and from studies whose data have been collected but not disseminated. Until these elements are present in the evidence base, no efficacy conclusion from any intranasal stem cell trial can be considered more than exploratory. Regardless of the numerically impressive changes within the groups.

This does not mean the field should stop. The safety signal across 98 analyzed participants is consistently favorable, and there are preliminary efficacy signals in cerebral palsy (Lv et al., 2023), perinatal stroke (Wagenaar et al., 2025), and Alzheimer’s disease (Xie et al., 2023) studies that are sufficiently interesting to justify well-designed follow-up investigation. The ALS trial (NCT06598202) is now recruiting with a quadruple-blind placebo-controlled design; the SO-ASC-INIVAT trial (NCT06858254) includes sham intranasal procedures in a crossover design; and the PASSIoN investigators have explicitly called for a powered randomized efficacy trial. These are the right next steps, and their timely completion and unconditional publication will determine whether intranasal stem cell therapy has a future in clinical medicine.

What this review makes clear is that the field currently stands at an evidentiary threshold. It has produced enough preliminary data to justify rigorous investigation, and enough risk bias documentation to define precisely what rigorous investigation must look like. The next decade of intranasal stem cell clinical research should be defined not by the accumulation of more open-label case series. It is essential that incomplete studies are executed and their results posted. The transparent publication of adequately powered, blinded, sham-controlled randomized trials with pre-registered endpoints, validated biological outcomes, and independent outcome assessments is needed.

This systematic review focused on available human trials, which demonstrated that intranasal stem cell administration is safe and feasible with potential therapeutic effects, but it has its limitations. With further clinical validation and regulatory progress, the intranasal route may emerge as a new and effective means of administration with precise targeting in regenerative medicine.

## Data Availability

The original contributions presented in the study are included in the article/supplementary material, further inquiries can be directed to the corresponding author.

## Glossary

IN: Intranasal
CNS: Central nervous system
BBB: Blood–brain barrier
MSC: Mesenchymal stem cells
NSC: Neural stem cells
iPSCs: Induced pluripotent stem cells
ESC: Embryonic stem cells
EVs: Extracellular vesicles
AD-MSC: Adipose-derived mesenchymal stem cells
BM-MSC: Bone marrow-derived mesenchymal stem cells
UC-MSC: Umbilical cord-derived regulatory T cells
bFGF: Basic fibroblast growth factor
EGF: Epidermal growth factor
HGF: Hepatocyte growth factor
VEGF: Vascular endothelial growth factor
KGF: Keratinocyte growth factor
IGF-1 and IGF-2: Insulin-like growth factors
MMPs: Matrix Metalloproteinases
PAIS: Perinatal arterial ischaemic stroke
AD: Alzheimer’s disease
PD: Parkinson’s disease
ALS: Amyotrophic lateral sclerosis
ADAS-Cog: Alzheimer’s Disease Assessment Scale-Cognitive Subscale
MMSE: Mini-Mental State Examination
PE: Pulmonary embolism
ARDS: Acute Respiratory Distress Syndrome
RCT: Randomized controlled trial
DLT: Dose-limiting toxicity
MTD: Maximum tolerated dose
NCI-CTCAE: National Cancer Institute Common Terminology Criteria for Adverse Events
